# *ImmRNA*: a database of RNAs associated with tumor immunity

**DOI:** 10.1093/database/baae052

**Published:** 2024-07-06

**Authors:** Fangfang Shen, Zelian Li, Pengfei Wu, Jinpeng Wang

**Affiliations:** Department of Infectious Diseases, The First Affiliated Hospital of Anhui Medical University, Hefei, Anhui 230027, China; Department of Infectious Diseases, Anhui Provincial Children’s Hospital, Hefei, Anhui 230022, China; Department of Obstetrics and Gynecology, The First Affiliated Hospital, Anhui Medical University, Hefei, Anhui 230020, China; Anhui Province Key Laboratory of Reproductive Health and Genetics, Hefei, Anhui 230020, China; Department of Neurosurgery, The First Affiliated Hospital of USTC, Division of Life Sciences and Medicine, University of Science and Technology of China, Hefei, Anhui 230001, P.R. China; Anhui Key Laboratory of Brain Function and Diseases, Hefei, Anhui 230001, P.R. China; Anhui Provincial Stereotactic Neurosurgical Institute, Hefei, Anhui 230001, P.R. China; Anhui Provincial Clinical Research Center for Neurosurgical Disease, Hefei, Anhui 230001, P.R. China; Anhui Province Key Laboratory of Translational Cancer Research, Bengbu Medical College, Bengbu, Anhui 233030, P.R. China; Department of Urology, The Second Affiliated Hospital of Harbin Medical University, Harbin 150086, China

## Abstract

The relationship between different ribonucleic acids (RNAs) and tumor immunity has been widely investigated. However, a systematic description of tumor immune-related RNAs in different tumors is still lacking. We collected the relationship of tumor immune-related RNAs from the published literature and presented them in a user-friendly interface, “*ImmRNA*” (http://www.immrna.cn/), to provide a resource to study immune-RNA-cancer regulatory relations. The *ImmRNA* contains 49 996 curated entries. Each entry includes gene symbols, gene types, target genes, downstream effects, functions, immune cells, and other information. By rearranging and reanalyzing the data, our dataset contains the following key points: (i) providing the links between RNAs and the immune in cancers, (ii) displaying the downstream effects and functions of RNAs, (iii) listing immune cells and immune pathways related to RNA function, (iv) showing the relationship between RNAs and prognostic outcomes, and (v) exhibiting the experimental methods described in the article. *ImmRNA* provides a valuable resource for understanding the functions of tumor immune-related RNAs.

**Database URL:**  http://www.immrna.cn/

## Introduction

Cancer is a complex and intricate disease, and the progressive accumulation of genetic and epigenetic alterations occurs in most human cancers (1–3). Substantial evidence has implicated that the occurrence of cancer is tightly associated with the microenvironment of tumors (4). The immune escape of tumor cells undermines the effectiveness of the immune response. Furthermore, many immune cells, such as tumor-associated macrophages, commonly promote tumor growth, angiogenesis, and metastasis (5–7). Many ribonucleic acids (RNAs), including coding RNAs and noncoding RNAs, have been proven to affect tumor development and treatment by modulating tumor immunity (8, 9). For instance, continuous activation of STAT3 inhibits antitumor immunity and mediates tumor-promoting inflammation (10). MALAT-1 is highly expressed in several human non-small cell lung cancer cell lines and displays an influential association with genes involved in cancer, mediating processes such as proliferation and immunity at the genetic level (11). However, pertinent information is fragmented and hidden in thousands of studies, which is inconvenient for researchers to systematically analyze these tumor immune-related RNAs, and databases integrating global relationships among cancer, immunity, and RNAs have rarely been reported.

Therefore, we rearranged and reanalyzed the collected data and finally integrated this relationship between RNA and tumor immunology. We completed a comprehensive review of eligible articles to gain a broad understanding of current RNA annotations. *ImmRNA* presents 25 856 curated RNAs, including 101 cancer types and 100 immune cell types. *ImmRNA* provides a sophisticated summary of tumor immunology and its associations with RNA dysregulation, thereby providing additional insights into tumorigenesis mechanisms and the treatment of cancers. We believe that *ImmRNA* can serve as a valuable resource for identifying and analyzing the functions of RNAs, especially immune-related RNAs, and advancing the development of human disease research in the era of precision medicine.

## Results

### Data collection and creation of the *ImmRNA*

**Figure 1.**
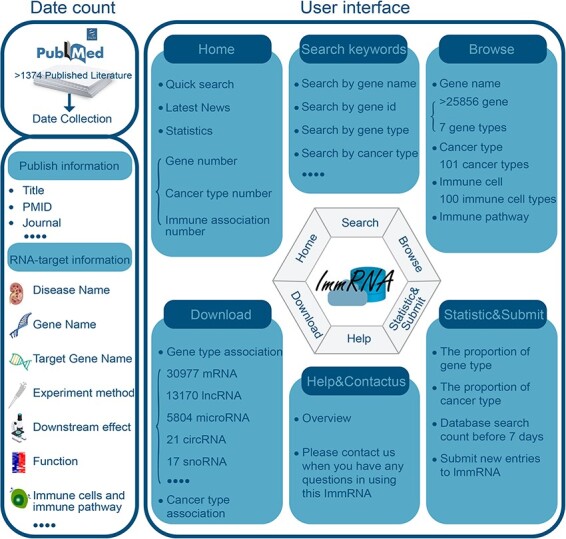
The workflow of the construction of the *ImmRNA*.

We found more than 50 000 articles that mentioned the keywords, which are present in the section of “Article search.” The literature with the above keywords was initially retrieved from the PubMed database. Then, we retained >5000 studies that included the relationships between RNAs and tumor immunology. We applied the following workflow to obtain specific information about RNAs and tumor immunology. First, we screened the literature based on the following criteria: experimental evidence (e.g. immunohistochemistry, immunofluorescence, RT‒qPCR, etc.) or nonexperimental evidence (e.g. GSEA, KEGG analysis, etc.) that could confirm the relationships between RNAs and tumor immunology. As a result, a total of 1374 studies were obtained according to our extraction requirements. Second, we carefully scrutinized the full text of the 1374 studies and obtained detailed information about RNAs and tumor immunology (Supplementary Table S1). We provided a concise and convenient web interface to enable the identification, browsing, and retrieval of immune- and cancer-associated RNAs and their annotation information. The *ImmRNA* interface allows users to freely search, download, and analyze datasets (Figure 1).

### Better identification of the tumor immune microenvironment

Many researchers have been studying the immunosuppressive tumor environment to identify ways to prevent tumor cells from evading immune attack. The role that immune-related RNAs play in biological processes has led to their gradual emergence as a research hotspot in recent decades. These studies mainly focused on the recognition and analysis of tumor immune-related RNAs. In total, *ImmRNA* contains 25 856 RNAs from 101 tumors and 41 tissues from humans. By directly searching for an immune-related RNA, users can visualize its function in a variety of tumors and its role in the tumor immune microenvironment, e.g. the effect on outcomes (e.g. poor, good), the immunological effect (e.g. immune cells, immune pathway, and immune activity), as well as the downstream effect. On the other hand, when researchers want to explore the role of the tumor immune microenvironment in certain tumors, they can search for keywords directly in the search box. Immune cells, immune pathways, and immune-related RNAs associated with the tumor will be presented on the search results page. Here is an example: when LUAD was searched in *ImmRNA*, a total of 2185 entries were obtained. Researchers can approximately evaluate which immune cells and immune pathways are associated with LUAD through macro search results. Users can also click on each entry to learn more about the immune-related RNAs, immune cells and immune pathways associated with LUAD. In brief, *ImmRNA* will help researchers better study the connections among immune-related RNAs, tumors, and immunity.

### Conclusion

Recently, tumor immunotherapy has greatly revolutionized the treatment of cancers and achieved remarkable outcomes (12). The tumor microenvironment (TME) is an important factor affecting tumor progression (13). More recent evidence suggests that the dysregulation of RNAs plays a significant role in cancer progression. In particular, dysregulated immune-related RNAs have an established role in cancer diagnostics, therapeutics, and prognostication. *ImmRNA* not only provides curated immune-RNA-cancer associations but also offers comprehensive insights into RNA functions in modulating the immune status of the TME. *ImmRNA* includes three distinctive features: (i) *ImmRNA* is a database of manually curated human tumor immunology-related RNAs with literature-supported evidence, (ii) *ImmRNA* integrates existing knowledge of the role of RNAs and immunity in tumor progression, which will further the investigation of treatments based on precision medicine, and (iii) *ImmRNA* provides a user-friendly and convenient website for browsing, searching, and downloading tumor immunology-related RNA data.

To provide consistency with the most recent studies, we will update *ImmRNA* every month. In the future, more additional functionalities and datasets will be uploaded into *ImmRNA*. We believe that *ImmRNA* will significantly improve our understanding of the role of RNAs in tumor immunology, and *ImmRNA* has the potential to be a readily available and valid resource for studying cancer.

## Methods

### Article search

We reviewed the immune-RNA-cancer regulatory relations that have been reported in published literature and created an interface that we named *ImmRNA*. To collect the maximal amount of information, we searched for published articles in the PubMed database with the following combination of keywords: “tumor or cancer, immune and lncRNA or long noncoding RNA,” “tumor or cancer, immune and microRNA or miRNA,” “tumor or cancer, immune and tRNA,” “tumor or cancer, immune and rRNA,” “tumor or cancer, immune and piRNA,” “tumor or cancer, immune and rRNA,” “tumor or cancer, immune and siRNA,” “tumor or cancer, immune and snRNA,” “tumor or cancer, immune and snoRNA,” “tumor or cancer, immune and SLRRNA,” “tumor or cancer, immune and SRPRNA,” “tumor or cancer, immune and tmRNA,” “tumor or cancer, immune and gRNA,” “tumor or cancer, immune and telomerase RNA,” and “tumor or cancer, immune and mRNA.”

### Data records

After reading the title and abstract, 1374 articles were deemed potential articles with the required data. After the full-text screening, the studies containing immune and cancer-associated RNAs and their detailed annotations were extracted, including the title, PubMed Identifier, publication date, tissue origin, cancer type, relationship between RNAs and prognostic outcomes, gene IDs, gene symbols, gene types, target genes, downstream effects, functions, experimental methods, relevant immune cells, and relevant immune pathways.

### Code availability

An mRNA is a series of specific datasets related to the study of the relationships among tumors, immunity, and RNAs. Technically, the website search provides global search functionalities and data analysis functionalities. In terms of the web architecture, we used a frontend web interface and backend separation technology. The application programming interface (API) was constructed using SpringBoot, and the web server was constructed using Vue. The server backend is responsible for providing data, and the web architecture is responsible for presenting data. The webserver was created using nginx (https://www.nginx.com/) to serve the interaction of frontend and backend, with an HTTP and reverse proxy to the backend. For data processing, we used the elasticsearch for lookup basic data, with MySQL (https://www.mysql.com) as the database system. In summary, we provided a user-friendly web interface that facilitates searching and exploring information in the database.

## Supplementary Material

baae052_Supp

## Data Availability

All data relevant to this study are incorporated into the article or available online in the *ImmRNA* (http://www.immrna.cn/).
